# Re: “American Thyroid Association and Thyroid Imaging Reporting and Data System developed by the American College of Radiology: which one is better at predicting malignancy risk?” in thyroidology

**DOI:** 10.1590/1806-9282.20231584

**Published:** 2024-05-03

**Authors:** Demet Sengul, Ilker Sengul, Tugrul Kesicioglu, Esma Cinar

**Affiliations:** 1 Giresun University, Faculty of Medicine, Department of Pathology – Giresun, Turkey.; 2 Giresun University, Faculty of Medicine, Division of Endocrine Surgery – Giresun, Turkey.; 3 Giresun University Faculty of Medicine, Department of General Surgery – Giresun, Turkey.

Dear Editor,

We read with a great deal and interest the research article entitled “American Thyroid Association and Thyroid Imaging Reporting and Data System developed by the American College of Radiology: which one is better at predicting malignancy risk?” by Andreda and colleagues^1^. This beneficial research of high quality seems to demand determining in order of comparing the capacity of the 2015 American Thyroid Association (ATA)^2^ and the 2017 American College of Radiology Thyroid Imaging Reporting and Data System (ACR-TIRADS)^3^ in predicting malignancy risk of thyroid nodules. We postulate that Andreda et al.^1^ performed a worthy comparison of ATA and ACR-TIRADS in terms of avoiding unnecessary fine needle aspiration (FNA) application, a valued and crucial issue in the thyroid lexicon, which has recently been published in the 67th volume of Rev Assoc Med Bras. However, we would like to emphasize some issues in thyroidology for the aforementioned study. First of all, thyroid nodules should be stated as a common clinical diagnostic challenge instead of a common clinical diagnosis (probably, a misspelling). Second, the authors reported the details of the ultrasound (a B-mode sonography) but not the size(s) and applying method(s) of the fine needles which had been used for the sampling procedures from the mentioned nodules to present them to the Department of (Cyto)Pathology. A wide range of (20–27 gauge in size) needles have been used for the procedure in different geographical regions (e.g., 25–27 gauge in most Western countries and 21–22 gauge in Japan)^4^. Debate is still ongoing on an optimal needle size for thyroid FNA cytology in thyroidology. In this sense, we reported a favorable non-diagnostic cytology rate on a sum of 500 nodules in 425 eligible consecutive outpatients during 38 months, involving ultrasonography (US)-guided FNA with a surgeon-performed US (SUS) in thyroid nodules with 27-G fine needles^5–12^. Therefore, would the outcomes of the study at that point be altered as they had harnessed significantly (i) finer or (ii) larger needle sizes? Herein, is it essential, at least, to state the relevant gauge(s) in order to design this deducing valued educational and technical study? Third, the authors emphasized that “the nodules with Category I, III, and IV, The Bethesda System for Reporting Thyroid Cytopathology (TBSRTC)^13^, were not included in the analysis due to the impossibility of assigning its behavior.” Nevertheless, they enunciated their purpose of comparing the capacity of the 2015 ATA^2^ and the 2017 ACR-TIRADS^3^ in predicting the malignancy risk of thyroid nodules^1^. Today, it has been widely accepted by thyroidologists that non-diagnostic (Category I) and indeterminate cytology (Categories III, IV, and V), in particular, TBSRTC, mostly deserve to be able to estimate an optimal and accurate malignancy risk. In addition, indeterminate cytology is constituted by Categories III, IV, and V^2^ but not “III and IV^1^”, TBSRTC^13^. Furthermore, Category V, *per se*, is not “malign” and even cannot “be considered malign”. Furthermore, mutational testing for BRAF or the seven-gene mutation marker panel (BRAF, RAS, RET/PTC, PAX8/PPARc) is recommended today in nodules suspicious for malignancy, Category V, cytology after consideration of clinical and sonographic features if such data would be expected to alter surgical decision-making^2^. *Breviter*, disorders with their diagnostic options of this papilionaceous and delicate endocrine gland remain their significance in tellurian^1–23^. As such, this work published in the 67th volume of Rev Assoc Med Bras^1^ is crucial for endocrine surgeons, endocrine pathologists, endocrinologists, head & neck surgeons and radiologists, otorhinolaryngologists, and thyroidologists, who stay informed of the growing spectrum of clinical management for challenging nodules for these thyroid providers and thyroid health as different peas in a pod^24–29^. This issue merits further investigation. We thank Andreda et al.^1^ for their valuable study.

## References

[1] Andrade MN, Costa JR, Sousa LM, Moreira LFGG, Oliveira RF, Álvares MCB (2023). American Thyroid Association and Thyroid Imaging Reporting and Data System developed by the American College of Radiology: which one is better at predicting malignancy risk?. Rev Assoc Med Bras (1992).

[2] Haugen BR, Alexander EK, Bible KC, Doherty GM, Mandel SJ, Nikiforov YE (2016). 2015 American Thyroid Association management guidelines for adult patients with thyroid nodules and differentiated thyroid cancer: the American Thyroid association guidelines task force on thyroid nodules and differentiated thyroid cancer. Thyroid.

[3] Tessler FN, Middleton WD, Grant EG, Hoang JK, Berland LL, Teefey SA (2017). ACR Thyroid Imaging, Reporting and Data System (TI-RADS): white paper of the ACR TI-RADS committee. J Am Coll Radiol.

[4] Maeda H, Kutomi G, Satomi F, Shima H, Mori M, Hirata K (2016). Clinicopathological characteristics of thyroid cancer misdiagnosed by fine needle aspiration. Exp Ther Med.

[5] Sengul I, Sengul D, Veiga ECA (2021). Revisiting optimal needle size for thyroid fine-needle aspiration cytology: not much finer, less non-diagnostic?. Rev Assoc Med Bras (1992).

[6] Sengul I, Sengul D (2022). Apropos of quality for fine-needle aspiration cytology of thyroid nodules with 22-, 23-, 25-, even 27-gauge needles and indeterminate cytology in thyroidology: an aide memory. Rev Assoc Med Bras (1992).

[7] Sengul D, Sengul I (2019). Association between Tsukuba elasticity scores 4 and 5 on elastography and Bethesda undetermined cytology on US-guided FNA with 27-G needle, verified by histopathology: a cut-off point of 20 mm of diameter designated for thyroid nodules. J BUON.

[8] Sengul D, Sengul I (2023). A vignette epexegesis of a model for training sonography-guided fine-needle aspirations in thyroidology and thyroidologists: think twice with needle size?. Rev Assoc Med Bras (1992).

[9] Sengul D, Sengul I (2022). Minimum minimorum: thyroid minimally invasive FNA, less is more concept? Volens nolens?. Rev Assoc Med Bras (1992).

[10] Sengul I, Sengul D (2021). Delicate needle with the finest gauge for a butterfly gland, the thyroid: is it worth mentioning?. Sanamed.

[11] Sengul I, Sengul D (2021). Proposal of a novel terminology: minimally invasive FNA and thyroid minimally invasive FNA; MIFNA and Thyroid MIFNA. Ann Ital Chir.

[12] Sengul I, Sengul D (2021). Big gain, no pain: thyroid minimally invasive FNA (Thy MIFNA): proposal of novelty in terminology. Rev Assoc Med Bras (1992).

[13] Cibas ES, Ali SZ (2017). The 2017 Bethesda system for reporting thyroid cytopathology. Thyroid.

[14] Sengul I, Sengul D (2021). Hermeneutics for evaluation of the diagnostic value of ultrasound elastography in TIRADS 4 categories of thyroid nodules. Am J Med Case Rep.

[15] Sengul D, Sengul I (2021). Reassessing combining real-time elastography with fine-needle aspiration biopsy to identify malignant thyroid nodules. Am J Med Case Rep.

[16] Sengul I, Sengul D (2022). Comment on: “Evaluating treatment options in managing thyroid nodules with indeterminate cytology of TBSRTC in thyroidology: addendum aut non?”. Rev Assoc Med Bras (1992).

[17] Sengul I, Sengul D (2021). Emphasis on the novel age cutoff, 55 years, for postsurgical adjuvant radioiodine as consideration for American Thyroid Association ¾ low-intermediate risk differentiated thyroid carcinoma. Rev Assoc Med Bras (1992).

[18] Sengul I, Sengul D (2020). Notes on “elastography for the diagnosis of high-suspicion thyroid nodules based on the 2015 American Thyroid Association guidelines: a multicenter study”. North Clin Istanb.

[19] Sengul D, Sengul I (2018). Is there any link between a kind of thyrocyte dysfunction, hypothyroidism, and inflammatory hematologic parameters in the cases having the benign thyroid nodules?: a 5-year single-centre experience. Sanamed.

[20] Sengul D, Sengul I (2018). Are there any variation in neutrophil lymphocyte ratio, mean platelet volume, and platelet count between papillary thyroid cancer and benign nodular thyroid diseases?. Sanamed.

[21] Sengul I, Sengul D (2021). Focusing on thyroid nodules in suspense: 10-15 mm with repeat cytology, category III, the Bethesda system for reporting thyroid cytopathology, TBSRTC. Rev Assoc Med Bras (1992).

[22] Sengul I, Sengul D (2021). Blurred lines for management of thyroid nodules in the era of atypia of undetermined significance/ follicular lesion of undetermined significance: novel subdivisions of categories IIIA and IIIB in a possible forthcoming the Bethesda system for reporting thyroid cytopathology, 3rd edition; amending versus unnecessary?. Rev Assoc Med Bras (1992).

[23] Sengul D, Sengul I, Soares JM (2022). Repercussion of thyroid dysfunctions in thyroidology on the reproductive system: conditio sine qua non?. Rev Assoc Med Bras (1992).

[24] Sengul I, Sengul D (2022). May 25-31, International Thyroid Awareness week & May 25, World Thyroid Day, 2022: indetermination of indeterminate cytology, AUS/FLUS, FN, SUSP, in thyroidology?. Sanamed.

[25] Sengul D, Sengul I (2023). World Thyroid Day 2023 in thyroidology: no overlook thyroid dis-eases to opt for “thyroid health” purposes. Rev Assoc Med Bras (1992).

[26] Sengul D, Sengul I (2023). Subdivision of intermediate suspicion, the 2021 K-TIRADS, and category III, indeterminate cytology, the 2017 TBSRTC, 2nd edition, in thyroidology: let bygones be bygones?. Ultrasonography.

[27] Sengul I, Sengul D (2023). The 2023 Bethesda system for reporting thyroid cytopathology: novi sub sole, subdivision is no more debatable, in thyroidology. Rev Assoc Med Bras (1992).

[28] Sengul I, Sengul D (2023). Evangely, the subcategorization has been announced in the 2023 Bethesda System for Reporting Thyroid Cytopathology: let bygones be bygones in Thyroidology!. Rev Assoc Med Bras (1992).

[29] Ali SZ, Baloch ZW, Cochand-Priollet B, Schmitt FC, Vielh P, VanderLaan PA (2023). The 2023 Bethesda system for reporting thyroid cytopathology. Thyroid.

